# Acquisition of methicillin-resistant *Staphylococcus aureus *after living donor liver transplantation: a retrospective cohort study

**DOI:** 10.1186/1471-2334-8-155

**Published:** 2008-11-11

**Authors:** Masao Hashimoto, Yasuhiko Sugawara, Sumihito Tamura, Junichi Kaneko, Yuichi Matsui, Junichi Togashi, Kyoji Moriya, Kazuhiko Koike, Masatoshi Makuuchi

**Affiliations:** 1 Artificial Organ and Transplantation Division, Department of Surgery, University of Tokyo, Tokyo, Japan; 2 Department of Infectious diseases, University of Tokyo, Tokyo, Japan

## Abstract

**Background:**

The incidence and risk factors of methicillin-resistant *Staphylococcus aureus *(MRSA) acquisition after living donor liver transplantation (LDLT) are unclear. The aim of the present study was to assess the incidence and to analyze the risk factors for the acquisition of MRSA after LDLT in adults by multivariate analysis.

**Methods:**

We retrospectively reviewed the data from 158 adult patients that underwent LDLT at the Tokyo University Hospital. The microbiologic and medical records of the patients from admission to 3 months after LDLT were reviewed. Uni- and multivariate analyses were performed to identify the risk factors for postoperative acquisition of MRSA.

**Results:**

Postoperative MRSA acquisition was detected in 35 of 158 patients by median postoperative day 18. Age (>= 60 y) and perioperative dialysis and/or apheresis predicted postoperative MRSA acquisition by multivariate analysis. In contrast, postoperative use of fluoroquinolone was negatively associated with acquisition of MRSA.

**Conclusion:**

MRSA arose early after LDLT in adults with a high incidence (35 of 158 patients). Surveillance culture should be checked periodically after LDLT to identify and prevent the transmission of MRSA.

## Background

Methicillin-resistant *Staphylococcus aureus *(MRSA) infection frequently complicates the postoperative course of deceased donor liver transplantation (DDLT) recipients [1-5]. In some centers, 91% (45 of 49 isolates) of all *Staphylococcus aureus *infections after DDLT are caused by MRSA [2].

Preoperative MRSA carriage is associated with an increased risk of MRSA infection after DDLT [1,3-5]. Positive MRSA culture in postoperative as well as in preoperative surveillance is important because the finding of MRSA colonization in a patient during hospitalization increases the risk of MRSA infection [6]. In one prospective study [6], the relative risk for developing MRSA infection in patients who had MRSA colonization was higher than that in patients who were not colonized with *Staphylococcus aureus*. In this particular study, 12 of 394 patients had MRSA colonization during hospitalization, and 4 of 12 (25%) later developed MRSA infection.

Few studies have focused on the factors associated with the acquisition of MRSA following liver transplantation. In one prospective study [7], the use of a urinary catheter for a prolonged period, postoperative bleeding at the surgical site, and preoperative use of fluoroquinolones independently increased the risk of MRSA colonization after DDLT. MRSA in cases of living donor liver transplantation (LDLT), in which operations are performed in a more scheduled manner, is not well documented.

The aim of the present study was to study the factors associated with the acquisition of MRSA after LDLT in adults assessed by surveillance cultures obtained from multiple sites, including nares, and to analyze the risk factors by multivariate analysis.

## Methods

### Patients

We retrospectively reviewed the data from 171 patients that underwent LDLT at the University of Tokyo Hospital, a 1150-bed teaching hospital, between August 2001 and November 2004. Of 171 patients, 13 were colonized with MRSA preoperatively and were excluded from the study. The median patient age was 51 years (range, 19–67). The indications for LDLT in these patients included hepatitis C (n = 53), hepatitis B (n = 24), primary biliary cirrhosis (n = 24), fulminant hepatitis (n = 18), biliary atresia (n = 8), autoimmune hepatitis (n = 7), primary sclerosing cholangitis (n = 5), metabolic disease (n = 5), alcoholic cirrhosis (n = 4), cryptogenic cirrhosis (n = 2), and others (n = 8). Of the 158 patients, 68 had hepatocellular carcinoma. The median Child-Pugh score and model for end stage liver diseases (MELD) score of those patients was 10 (range, 5–14) and 13 (range, -3 to 48), respectively. The microbiologic and medical records of the patients from admission to 3 months after LDLT were reviewed. The present study was approved by The University of Tokyo Ethical Committee. The data used for the study are publicly available.

### Donor selection

Donors were selected from the patients' relatives. Age, blood type, graft size, and liver function were also taken into consideration. ABO blood groups were required to be identical to or compatible with that of the recipients. The graft type was determined according to the ratio of the estimated graft volume to the recipient's standard liver volume ratio [8,9]. Our surgical technique for recipient and donor surgery is described elsewhere [10]. Donors were not routinely screened for *Staphylococcus aureus *perioperatively.

### Perioperative management

Antimicrobial prophylaxis consisted of intravenous cefotaxime (1.0 g just before surgery, followed by 1.0 g every 6 hours intraoperatively and thereafter), ampicillin/sulbactam (1.0 g just before surgery, followed by 1.5 g every 12 hours intraoperatively and thereafter), and gentamicin, 60 mg every 12 hours after surgery) for 5 days.

To prevent fungal infection, fluconazole (200 mg every 24 hours) was administered intravenously for 7 days after surgery. All patients received the same immunosuppressive regimens using tacrolimus (Prograf, Astellas Pharmaceutical Corporation, Tokyo, Japan) and methylprednisolone (Solu-Medrol, Pfizer Inc., New York, NY). The details of the regimen are reported elsewhere [11].

### Definition of MRSA colonization

All the patients were screened preoperatively for *Staphylococcus aureus *on admission for LDLT. Follow-up specimens were collected twice a week during the first month after LDLT, and thereafter once a week during the hospital stay. Routine surveillance specimens consisted of swabs of the anterior nares, pharynx, sputum, urine, and stool. In addition, swabs of wound or skin lesions, bile, and discharge from the abdominal cavity were collected postoperatively. Blood samples, collected percutaneously, and a segment of a removed intra-vascular catheter were also submitted when infection was suspected as the followings: fever (> 38°C), chills, or hypotension. Other clinical samples were added in patients with suspected infection according to the discretion of the attending physician.

Specimens were plated onto mannitol-salt agar or sheep blood agar. *Staphylococcus aureus *was identified using standard microbiologic methods.

Methicillin resistance was determined using a disk diffusion test performed on Mueller-Hinton agar after incubation for 24 to 48 hours at 30°C. By the microdilution method, strains with an oxacillin minimum inhibitory concentration value of at least 4 μg/ml were defined as MRSA [12]. Patients colonized with *Staphylococcus aureus *at any site, and at any time during the hospital stay, were considered carriers.

### Definition of MRSA infection

Nosocomial infections were defined according to the reports from the Centers for Disease Control and Prevention in 1988 and in 1992, as described elsewhere [13,14]. When MRSA was isolated from culture samples in the presence of nosocomial infection and other pathogenic organisms were absent, MRSA infection was diagnosed.

### Management of precaution for transmission of MRSA

0.2% benzalkonium chloride ethanol solution (Welpas, Maruishi Pharmaceutical Corporation, Oosaka, Japan) were used for hand hygiene of patients, medical, and non-medical staffs in contact with patients. Contact precautions were taken in cases with MRSA colonization and/or infection. Eradication therapy such as intranasal mupirocin was not routinely performed. The screening of medical staffs for detection of MRSA was not also performed during the study period.

### Background and clinical data collection

Background and clinical data collected for each patient included:

1) preoperative variables (age, gender, etiology of the underlying liver disease, presence of hepatocellular carcinoma, Child-Pugh score, MELD score, presence of ascites, use of diuretics, presence of encephalopathy, the international normalized ratio of prothrombin time level, serum bilirubin level [mg/dl], serum albumin level [g/dl], serum creatinine level [mg/dl], use of steroid, use of antimicrobials during the month before LDLT, presence of diabetes mellitus, history of hospital stay during the 6 months before LDLT, and methicillin-susceptible *Staphylococcus aureus *colonization;

2) surgical variables (operation time [hours], estimated blood loss [ml], blood transfusion [ml], graft volume/standard liver volume ratio [%], and application of duct to duct biliary reconstruction;

3) postoperative variables (length of urinary catheter insertion [days], length of arterial catheter insertion [days], length of central venous catheter insertion [days], length of endotracheal tube insertion [days], necessity for reoperation, acute rejection, cytomegalovirus infection, fungal infection, and postoperative use of antimicrobials other than the routine perioperative prophylaxis); and

4) pre- and postoperative variables (length of intensive care unit stay [days], and application of dialysis and/or apheresis).

### Statistical analysis

Quantitative variables are presented as median and range. Categorical variables are presented as absolute counts. Univariate analysis was used to identify associations between each of the variables recorded and postoperative acquisition of MRSA. Wilcoxon rank sum test was used to compare the quantitative variables. Chi-square test or Fisher's exact test was used to compare the categorical data.

For multivariate analysis, only variables with a *p *value of less than 0.25 in the univariate analysis were entered into a logistic regression model by the backward-elimination procedure. The final regression model included covariates associated with a likelihood ratio of *p *less than 0.15. The results of the logistic regression were reported as odds ratios with 95% confidence intervals. A *p *value of less than 0.05 was considered statistically significant. All statistical analyses were performed using the JMP5.1 software package (SAS Institute Inc., Cary, NC).

## Results

### Acquisition of MRSA after LDLT

**Figure 1:**
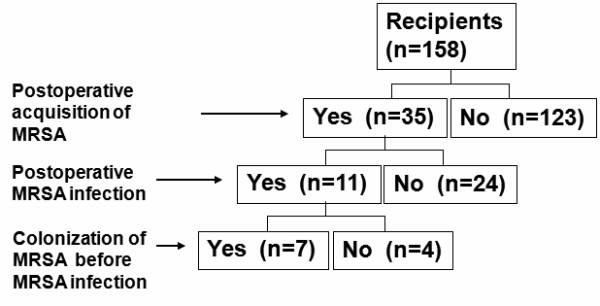
The patient profile of postoperative MRSA colonization and infection Abbreviations: MRSA, methicillin-resistant *Staphylococcus aureus*; LDLT, living donor liver transplantation.

The median number of screening samples for each patient and the compliances with surveillance culture for nares, pharynx, sputum, urine, and stool were 9 (range, 1–25), 9 (range, 0–25), 5 (range, 0–25), 9 (range, 1–24), and 6 (range, 0–22) samples, and 82%, 82%, 50%, 80%, and 60%, respectively. Data on the detection of postoperative acquisition of MRSA are summarized in Figure 1. Postoperative acquisition of MRSA was detected in 35 of 158 patients (22%) during the study period. The median period of time between LDLT and detection of MRSA was postoperative day 18 (range, 1–89 days). In 8 of 35 patients, MRSA was detected during the intensive care unit stay. Median length of hospital stay after LDLT were 45 (range, 6–90) days in patients without MRSA acquisition and 59 (range, 33–90) in those with MRSA acquisition, respectively (*p *= 0.0006). Eleven of 158 (7%) patients developed MRSA infection during the study period: deep incisional surgical site infection (SSI) in 5, organ/space SSI in 2, intraabdominal infection in 2, lower respiratory infection in 1, and primary bloodstream infection in 1 patient, respectively. MRSA infections were eventually diagnosed in 7 of 31 patients (23%) who were colonized with MRSA while only 4 of 127 subjects (3%) with negative surveillance cultures developed MRSA infection (p = 0.01). Table 1 shows the frequency of detection of MRSA from different clinical and surveillance specimens. In 30 subjects (86%) MRSA was isolated in more than 2 sites.

**Table 1:** Frequency of MRSA detection in different surveillance and clinical specimens of 35 patients

Sites	Number of patients (%)
Nares	24 (69%)
Pharynx	21 (60%)
Sputum	18 (51%)
Stool	18 (51%)
Urine	11 (31%)
Wound	8 (23%)
Intraabdominal drain	5 (14%)
Bile	3 (9%)
Intravascular catheter	2 (6%)
Ascites	2 (6%)
Pleural effusion	2 (6%)
Blood	1 (3%)

Thirty of 35 patients had MRSA detected from multiple sites.

### Risk factors for the Acquisition of MRSA after LDLT

The results of the univariate analyses assessing the association between the acquisition of MRSA and clinical covariates are shown in an additional file 1. Age of at least 60 years (*p *= 0.01), presence of an endotracheal tube for at least 3 days (*p *= 0.03), and perioperative dialysis and/or apheresis (*p *= 0.008) were significant factors affecting the acquisition of MRSA. In the multivariate analyses (Table 2), 10 risk factors with a univariate *p *value of less than 0.25 were entered into a logistic regression model by the backward-elimination procedure. In the final model, age of at least 60 years and perioperative dialysis and/or apheresis predicted the postoperative acquisition of MRSA. In contrast, postoperative use of fluoroquinolone was negatively associated with acquisition of MRSA.

**Table 2:** Multivariate analysis of risk factors for the acquisition of MRSA after LDLT

Variable	Odds Ratio (95% Confidence interval)	*p *Value
Age >= 60	3.33(1.17–9.58)	0.03
Duct to duct biliary reconstruction	3.18(0.92–15.22)	0.07
Endotracheal tube (day) >= 3	2.26(0.87–5.84)	0.09
Postoperative use of beta lactam	0.49(0.20–1.23)	0.13
Postoperative use of fluroquinolone	0.14(0.007–0.88)	0.03
Perioperative dialysis and/or apheresis	2.92(1.16–7.39)	0.02

Abbreviations: MRSA, methicillin-resistant *Staphylococcus aureus*; LDLT, living donor liver transplantation.

## Discussion

To date, this is the largest series study of the presence of MRSA after LDLT in adults. Of 158 patients, 35 (22%) presented a positive culture for MRSA by median postoperative day 18. The rate in the present study was higher than that in the recently published prospective study in DDLT [7], in which 9 of 60 (15%) patients acquired nasal MRSA colonization by median postoperative day 24. Patients who acquired MRSA were significantly associated with an increased length of hospital stay in the present study. Similary, Singh et al. reported that an increased length of hospital stay was associated with new Staphylococcus aureus carriage acquisition in DDLT[15]. Longer hospital stay, which is one of a marker for greater severity of illness, also might have identified high risk candidates requiring more intensive care, which could lead an increasing chance of MRSA transmission. Although the anterior nares is the most frequent carriage site for *Staphylococcus aureus *[16], other extra-nasal sites such as skin, perineum, pharynx, gastrointestinal tract, vagina, and axillae can harbor the organism [5,16]. MRSA from the nares was detected in 24 of 158 (15%) patients in the present study, which was comparable to the result of the previous report [7]. Of the 31 patients with MRSA-positive cultures, 7 (23%) subsequently developed MRSA infection. In addition, 7 of 11 (64%) patients who developed MRSA infection were colonized with MRSA prior to infection while only 4 of the 127 subjects (3%) with negative surveillance cultures developed MRSA infection (p = 0.001). This is in line with the findings of other authors, which indicated that interventions aimed at curtailing the transmission of MRSA may have a beneficial impact on the incidence of MRSA infection [17,18]. Furthermore, this is useful information in that it allows for earlier administration of a more appropriate antibiotic such as vancomycin in patients suspected of having MRSA infection.

The present study indicated that age of at least 60 years increased the risk of postoperative acquisition of MRSA by multivariate analysis. As a large number of variables (N = 39) were included in the analyses in the present study, we must recognize the possibility that statistical association might have occurred by chance. The exact reason why older patients acquire MRSA more frequently after LDLT is unclear. Some previous studies [19,20] reported that older age was a risk factor for MRSA acquisition during hospitalization, although the interpretation was not described.

Another risk factor indicated by the present study, dialysis and/or apheresis, requires indwelling devices such as intravascular catheters. Invasive procedures are "entrance gates" for microorganisms, and potential hand contamination of personnel who perform these procedures might increase the risk of MRSA transmission [19]. On the other hand, perioperative dialysis and/or apheresis might merely be suggestive of the intensity of care required for patients in the present study. Perioperative dialysis and/or apheresis, mostly indicated in cases of deteriorated liver dysfunction in the present study, might suggest the deteriorated general conditions of patients, making them more prone to infectious diseases. Intensity of care required can be considered a surrogate marker for a number of manipulations that are major risk factors for MRSA transmission [21].

It might be better to adopt additional strategies for patients with these risk factors of MRSA acquisition. Singh et al. [15] reported an impact of an aggressive infection control strategy on *Staphylococcus aureus *infection in liver transplant recipients, including use of surveillance cultures to detect nasal and rectal colonization, use of cohort and contact isolation precautions, decolonization with intranasal mupirocin therapy, and educating patients and visitors about hand hygiene and MRSA transmission. In that study, the rate of new acquisition of *Staphylococcus aureus *decreased from 46% during the pre-intervention period to 10% during the post-intervention period, and the rate of *Staphylococcus aureus *infection decreased from 40% to 4%, respectively.

The intensity of the use of antimicrobials, measured by the presence of preoperative antibiotic use during the month before LDLT did not correlate with the acquisition of MRSA after transplantation in the present study. Furthermore, postoperative use of fluoroquinolone was negatively associated with acquisition of MRSA, which was contrary to our expectations. It was difficult to analyze whether the postoperative frequency of use of antimicrobials increased a risk of MRSA acquisition in the present study. Only the antimicrobials used before the first date of detection of MRSA were included in the analysis, which caused the difference of observation period for exposure to antimicrobials between patients with and without acquisition of MRSA. The median period of time between LDLT and detection of MRSA was postoperative day 18, and 17 of 35 (49%) patients acquired MRSA within 2 weeks after the operation. Although there is little doubt that widespread use of antimicrobials provides multidrug-resistant strains of MRSA with a selective survival advantage [22], the relation between MRSA and antimicrobials seems more complex in the current series. Some studies [23-29] failed to show such an association by multivariate analysis. In other studies [30-33], exposure to specific antimicrobials, such as third generation cephalosporins, amoxicillin with clavulanic acid, quinolones, and other broad-spectrum antibiotics, increased the risk of MRSA infection or colonization. Crowcroft et al. [33] found no association between total antimicrobial use and MRSA colonization or infection and suggested that the problem was the inappropriate use of antimicrobials, not excessive use. This discrepancy is probably due to the fact that in the present study all the patients received long courses of multiple antimicrobials resulting in broad coverage, as perioperative prophylaxis per protocol, and it is therefore difficult to detect the effect of a specific antimicrobial. It might also be better for reducing the acquisition of MRSA to shorten prophylactic use of antimicrobials to a maximum of 48 hours as used in other transplant centers [7,34].

One limitation of the present study is that the MRSA carriage pattern was not analyzed. Longitudinal studies have distinguished three *Staphylococcus aureus *carriage patterns in healthy individuals [15,35]. This distinction is important because persistent carriers have higher *Staphylococcus aureus *loads and a higher risk of acquiring *Staphylococcus aureus *infection [36].

Another limitation of the present study is that we could not differentiate the specific MRSA strains. Pulsed-field gel electrophoresis analysis was not accessible and the data were not obtained. Therefore, we could not analyze the impact of MRSA transmission, such as patient-to-patient transmission by transient carriage on the hands of the medical staff in detail. Our observation remains speculative on this point. Similarly, it was impossible to know whether infection was due to the same strain as that of the colonization or to a newly acquired strain. Chang et al. [4] analyzed the isolates from infected sites and from the anterior nares in seven patients with MRSA infection, and reported that the same isolates were detected. Such detailed analyses might yield further information to prevent the spread of MRSA following LDLT.

## Conclusion

There is a high incidence of MRSA early after LDLT in adults. Surveillance cultures should be performed periodically after LDLT to identify and prevent the transmission of MRSA.

## Abbreviations

LDLT: living donor liver transplantation; DDLT: deceased donor liver transplantation; MELD: model for end stage liver diseases; MRSA: methicillin-resistant *Staphylococcus aureus*; MSSA: methicillin-susceptible *Staphylococcus aureus*.

## Competing interests

The authors declare that they have no competing interests.

## Authors' contributions

MH, YS, and MM designed the Research project and gave a critical view of manuscript writing. JK, YM, JT, KM, and KK helped in collecting the specimens, and the microbiologic and medical records. MH, YS, and ST wrote the manuscript. All the authors have read and approved the final manuscript.

## Pre-publication history

The pre-publication history for this paper can be accessed here:



## Supplementary Material

Additional file 1 **An additional table.** Association between postoperative acquisition of MRSA and perioperative variables by the univariate analysis. Click here for file
