# Supplementary material for: Acquisition of methicillin-resistant Staphylococcus aureus after living donor liver transplantation: a retrospective cohort study
Source: BMC Infect Dis. 2008 Nov 11;8:155. doi: 10.1186/1471-2334-8-155 (PMC2625350; doi:10.1186/1471-2334-8-155)
Supplement: Additional file 1 — An additional table. Association between postoperative acquisition of MRSA and perioperative variables by the univariate analysis. [file 1471-2334-8-155-S1.doc]

An additional table. Association between postoperative acquisition of MRSA and perioperative variables by the univariate analysis

| Variables | Acquisition of MRSA (-) (n=123) | Acquisition of MRSA (+) (n=35) | *p* Value |
| --- | --- | --- | --- |
| Preoperative variables |  |  |  |
| Age(yr)  >= 60 | 50 (19-66)  13(11%) | 55 (24-67)  10(29%) | 0.04  0.01 |
| Gender, male/female | 71/52(58%/42%) | 23/12(66%/34%)) | 0.44 |
| Underlying Liver disease |  |  |  |
| Hepatitis C | 38(31%) | 15(43%) |  |
| Hepatitis B | 22(18%) | 2(6%) |  |
| Primary biliary cirrhosis | 20(16%) | 4(11%) |  |
| Fulminant hepatitis | 11(9%) | 7(20%) |  |
| Biliary atresia | 6(5%) | 2(6%) |  |
| Autoimmune hepatitis | 6(5%) | 1(3%) |  |
| Primary sclerosing cholangitis | 5(4%) | 0(0%) |  |
| Metabolic disease | 5(4%) | 0(0%) |  |
| Alcoholic cirrhosis | 1(1%) | 3(9%) |  |
| Cryptogenic cirrhosis | 2(2%) | 0(0%) |  |
| Others | 7(6%) | 1(3%) |  |
| Hepatocellular carcinoma | 40(33%) | 14(40%) | 0.43 |
| Child-Pugh score  >= 10 | 10 (5-14)  63(51%) | 10 (6-13)  19(54%) | 0.73  0.85 |
| MELD score  >= 15 | 12.6 (-3.4-48.2)  37(30%) | 14.4 (4.3-41.6)  11(31%) | 0.33  1.0 |
| Ascites | 57(46%) | 15(43%) | 0.85 |
| Use of Diuretics | 67(55%) | 15(43%) | 0.25 |
| Encephalopathy | 19(15%) | 9(26%) | 0.21 |
| PT-INR  >= 1.7 | 1.65 (1.02-7.48)  53(43%) | 1.63 (1.14-2.35)  14(40%) | 0.70  0.85 |
| Serum bilirubin (mg/dl)  >= 10.0 | 3.6(0.3-38.6)  27(22%) | 3.8(1.0-32.4)  11(31%) | 0.26  0.27 |
| Serum albumin (mg/dl)  >= 2.5 | 2.9(1.5-4.4)  108(88%) | 2.9(2.3-4.0)  27(77%) | 0.73  0.10 |
| Serum creatinine (mg/dl)  >= 1.5 | 0.7(0.3-2.7)  7(6%) | 0.7(0.4-7.7)  3(9%) | 0.49  0.69 |
| Use of steroid | 12(10%) | 3(9%) | 1.0 |
| Preoperative use of antimicrobials | 34(28%) | 9(26%) | 1.0 |
| Beta lactam | 34(28%) | 8(23%) | 0.67 |
| Fluroquinolone | 8(7%) | 3(9%) | 0.71 |
| Amynoglycoside | 1(1%) | 1(3%) | 0.40 |
| Diabetus mellitus | 15(12%) | 8(23%) | 0.17 |
| History of hospital stay (the past 6 months) | 98(80%) | 30(86%) | 0.48 |
| MSSA colonization | 58(47%) | 19(54%) | 0.57 |
| Surgical variables |  |  |  |
| Operation time (hr)  >= 16 | 14.9(10.7-25.5)  36(29%) | 14.9(11.1-22.2)  13(37%) | 0.80  0.41 |
| Blood loss (ml)  >=8000 | 5240(890-53835)  34(28%) | 5775(1710-34800)  12(34%) | 0.29  0.53 |
| Blood transfusion (ml)  >= 8000 | 7160(900-42890)  50(41%) | 7220(4240-30600)  15(43%) | 0.44  0.85 |
| GV/SLV ratio (%)  >= 40 | 48(32-71)  103(84%) | 46 (25-75)  30(86%) | 0.80  1.0 |
| Duct to duct biliary reconstruction | 95(77%) | 32(91%) | 0.09 |
| Postoperative variables |  |  |  |
| Urinary catheter (day)  >=14 | 9(3-90)  43(35%) | 10(3-73)  16(46%) | 0.94  0.32 |
| Arterial catheter (day)  >=5 | 4(1-70)  61(50%) | 4(1-34)  17(49%) | 0.79  1.0 |
| Central venous catheter (day)  >=14 | 8(3-78)  19(15%) | 8(3-68)  8(23%) | 0.51  0.32 |
| Endotracheal tube (day)  >=3 | 1(1-69)  26(21%) | 2(1-33)  14(40%) | 0.03  0.03 |
| Reoperation | 32(26%) | 11(31%) | 0.53 |
| Acute rejection | 24(20%) | 9(26%) | 0.48 |
| Cytomegalovirus infection | 46(37%) | 10(29%) | 0.33 |
| Fungal infection | 4(3%) | 1(3%) | 1.0 |
| Postoperative use of antimicrobials^1^ | 92(75%) | 21(60%) | 0.09 |
| Beta lactam^1^ | 92(75%) | 21(60%) | 0.09 |
| Fluroquinolone^1^ | 14(11%) | 1(3%) | 0.19 |
| Amynoglycoside^1^ | 14(11%) | 2(6%) | 0.53 |
| Pre- and postoperative variables |  |  |  |
| ICU stay (days)  >= 7 | 5(3-42)  22(17%) | 5(1-12)  7(20%) | 0.80  0.81 |
| Perioperative dialysis and/or apheresis | 26(21%) | 16(46%) | 0.008 |

Abbreviations: MRSA, methicillin-resistant *Staphylococcus aureus*; PT-INR, the international normalized ratio of prothrombin time; MSSA, methicillin-susuceptible *Staphylococcus aureus*; GV, graft volume; SLV, standard liver volume; ICU, intensive care unit.

1. Only the antimicrobials other than the routine perioperative prophylaxis, which were used before the first date of acquisition of MRSA, were included.
